# Uncorking Haloanisoles in Wine

**DOI:** 10.3390/molecules28062532

**Published:** 2023-03-10

**Authors:** Abigail Keng, Andreea Botezatu

**Affiliations:** Department of Horticultural Sciences, Texas A&M University, College Station, TX 77843, USA

**Keywords:** haloanisole, wine, 2,4,6-trichloroanisole (TCA), 2,4,6-tribromoanisole (TBA), 2,3,4,6-tetrachloroanisole (TeCA), pentachloroanisole, halophenol

## Abstract

Haloanisoles in wine have devastating effects on the aroma and quality of the wine. 2,4,6-trichloroanisole (TCA) was discovered and coined as “cork taint” in 1982. However, we now understand that there are many more haloanisoles that contribute to these musty odors, including 2,4,6-Tribromoanisiole (TBA), 2,3,4,6-tetrachloroanisole (TeCA), and pentachloroanisole (PCA). While TCA, TeCA, and PCA can all be traced back to the cork, TBA’s phenol precursor is ubiquitous in building material as a fire retardant, making it a much larger vector. All haloanisoles have the ability to aerosolize and resettle onto surfaces in the winery, making this a very difficult problem to eliminate. This literature review will cover the multiple haloanisoles found in wine, their sensory impacts, their effect on wine quality, and current methodologies with regard to their analysis.

## 1. Introduction

In 1982, 2,4,6-trichloroanisole (TCA) was the first haloanisole identified as causing musty odors in wine. Even before its discovery, wine makers suspected that corks had something to do with the odor, thus naming the fault that leads to musty odors in wine “cork taint”. This groundbreaking research also concluded that in some wines the concentration of TCA did not correlate with the level of musty odors the wine had [1]. We now know that there are multiple haloanisoles that can be the cause of musty odors in wine, including 2,4,6-tribromoanisole (TBA), 2,3,4,6-tetrachloroanisole (TeCA), and pentachloroanisole (PCA) [2]. Despite this knowledge, most of the literature solely focuses on TCA as the main cause of cork taint, in spite of the multitude of vectors which the other haloanisoles possess to contaminate wine.

This literature review will cover the multiple haloanisoles found in wine, their sensory impacts, their effect on wine quality, and current methodologies with regard to their analysis.

## 2. What Are Haloanisoles in Wine?

Haloanisoles in wine all cause the same musty and moldy odors and are indistinguishable from each other in a wine matrix [3]. Although detrimental to wine quality, haloanisoles are not toxic; however, their halophenol precursors are “highly toxic” [1]. The main difference between the haloanisoles are their sources and how they come into contact with wine.

### 2.1. Trichloroanisole

Trichloroanisole was the first haloanisole identified as the causal compound for musty, moldy aromas in wine in 1982 and is the most well-researched haloanisole in wine. Previously, TCA was only studied as an off flavor in chicken eggs and broilers [1]. TCA was originally found in corks that had been bleached with chlorine bleach in order to create a uniform white color for the corks [1]. The chlorine would mix with the natural fungus present in the cork and produce TCA. The TCA would then be released into the wine once it was sealed and left to age in bottle. Buser proposed that the “replacement of chlorine treatment in the processing of cork” would fix this issue. The industry has since eradicated the use of chlorine bleaching of corks, so how can we still have “corked” wines? Another change that cork producers made after becoming aware of TCA is not washing the corks using public water that contains chlorine for human health and safety [4]. Wineries now also know to dechlorinate the water that they use for wine making if they are using public water.

Studies conducted in cork tree forests in Portugal indicated the presence of TCA in the bark. Further research showed a higher concentration of TCA at the base of the trees. The researchers hypothesized that the past use of chlorophenol-based biocides, containing either TCP or PCP, led to these compounds being still present, even though the spraying regime was stopped in the 1980’s [4]. In 2005, a study conducted by Herve found a 76% decrease of ‘releasable TCA’ in corks. He defined releasable TCA as the amount of TCA able to move from the cork into a solution such as wine. It is possible that in the past 17 years since the study conducted by Herve, this amount of releasable TCA has decreased even more.

**Figure 1:**
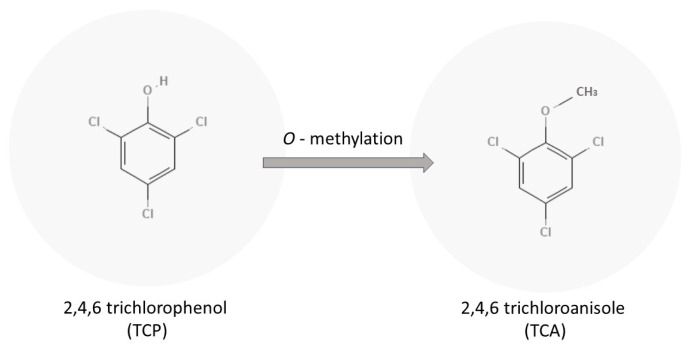
Microbiological formation of TCA by O-methylation of TCP.

The precursor to TCA is 2,4,6-trichlorophenol or TCP. TCP can be methylated by different fungi to produce TCA as shown in Figure 1. Different fungi showed varying aptitudes to transform the TCP to TCA. *Trichoderma* and *Fusarium* were found to be strong methylators with a 25% conversion of TCP to TCA, and *Penicillium*, *A. strictum*, *C. sitophila*, and *C. oxysporum* were found to be medium methylators, with only a 5–10% transformation of TCP into TCA [1]. A recent study in 2021 found that *Aspergillus versicolor* and *Paecilomyces variotii* were found to be strong methylators and are very common in wooden constructions [5].

### 2.2. Tribromoanisole

There are many sources in the winery for tribromoanisole and its precursor tribromophenol. Fireproofing agents can contain tribromoanisole or tribromophenol (TBP); these agents can become aerosolized, contaminate wood or other products treated with brome fumigates, and can spread to all parts of the winery [2,6].

Another source of bromine-based fire retardants is spray foam insulation. By itself, spray foam insulation is flammable; however, when bromine flame retardants are added they “enhance the flame retardancy of the foams, helping to inhibit ignition or slow down the process of combustion” [7]. According to the FDA, spray foam insulation can be installed and left unexposed—not covered by drywall, for example. Older bromine-based fire retardants such as Hexabromocyclododecane (HBCD) are being phased out; however, newer ones are being implemented.

Methyl Bromide is a chemical fumigant that is used to treat shipping containers, wood products, and other materials in order to protect them from invasive pests. It is also a substance that is being phased out of use by the Environmental Protection Agency (EPA) due to its damaging effects on the atmosphere [8]. 

Recently, a study showed that there are still measurable concentrations of fumigants in both treated and untreated containers. These concentrations are higher in containers that are treated with methyl bromide and in containers that contain metal or glass items, as well as containers from China [9]. 

Methyl bromide is still in use for wood treatment in order to adhere to the International Standards for Phytosanitary Measures. These measures are necessary to prevent the introduction of harmful species that could potentially disrupt our ecosystems [10]. 

TBP can become TBA by O-methylation of fungus, such as *Paecelomyces variotii*, which has also been found to convert TCP into TCA [11].

A study performed in 2004 tested three wineries for TBA contamination. The authors sampled a variety of places, including the atmosphere, wines stored in stainless steel vs. barrel, walls of the buildings, and barrels of different ages. All wineries had TBA present, ranging in concentration from 2–2185 ng/L [12].

TBA can become aerosolized and resettle onto many things in the winery and lead to drastic quality loss in wines.

### 2.3. Tetrachloroanisole & Pentachloroanisole

Tetrachloroanisole (TeCA) and pentachloroanisole (PCA) are unique haloanisoles due to the co-use of their phenol precursors—2,4,6-tetrachlorophenol (TeCP) and pentachlorophenol (PCP)—in various applications. PCP was once the most widely used biocide in the United States, but its use has since been heavily restricted by the EPA. Despite this, PCP is still used to preserve utility poles and railroad ties. TeCP is often found in combination with PCP, as it is used as a component of PCP based biocides. This means that when one of the compounds is detected, it is likely that the other is present as well [13]. Due to its previous use as a biocide in the United States, residual PCP and TeCP can be found in oak forests used to create many products found in the winery. PCP and TeCP are easily methylated by fungi found in the air that includes, but are not limited to, *Trichderma virgatum, Aspergillus sydowi, Scopulatriopsis brevicaulis, and penicillium* spp. [14]. Both the phenol and the anisole can become airborne and resettle in and on soil, water, trees, etc., thus making it extremely difficult to be completely eliminated in natural settings. Wood is commonly used in wineries, from the building structure to wood pallets, barrels, and more. Though it may be a useful material, it is important to consider the potential for contamination from TeCA and PCA, as well as their phenol precursors.

## 3. Sensory Characteristics and Thresholds

The presence of haloanisoles in wine can have a strong negative effect on its aroma, producing unpleasant odors such as musty, moldy, wet dog, wet newspaper, or damp basement [6]. Haloanisoles are found in very low concentrations, in the nanograms per liter (ng/L) or parts per trillion (ppt) range. The presence of these compounds in wine can cause a noticeable decrease in its flavor, aroma, and overall quality.

Human sensory detection of haloanisoles in wine can be influenced by several variables. Sensory evaluations previously conducted reported varying thresholds, most of those ranging between 3–10 ng/L for TCA and TBA wine [15,16]. Detection thresholds were reportedly lower, about 3–4 ng/L, for trained panelists as well as people who have knowledge of wine faults [17,18]. Detection thresholds are also lower in white wines than red wines; this can be attributed to the increased complexity of red wines due to higher tannin concentrations and masking effects generated by oak aging [17].

Most threshold detection studies, whether using a trained or untrained panel, have only looked at trichloroanisole and its effect on wine sensory profiles. However, one study reports the tribromoanisole threshold to be between 2–7.9 ng/L in wine [6]. Trichloroanisole and tribromoanisole have also been identified as olfactory suppressors. Small amounts of trichloroanisole and tribromoanisole are able to suppress channels in olfactory cilia, causing odor losses and reduction of flavors. [18]. This more recent research might help explain the findings of Prescott et al., 2004, who showed that a minority of people could not detect TCA when wines were spiked with high levels of it. It is perhaps as likely that TCA and TBA act as sensory suppressors, which reduces their perceptibility, as some people being insensitive to these compounds [18]. This highlights an important factor in the production of wine, as the presence of these odor suppressors can drastically reduce the quality and taste of the finished product.

Other haloanisoles, including PCA and TeCA, are not often included in these sensory experiments. Odor thresholds for TeCA have been reported between 5–15 ng/L and for PCA 10,000 ng/L in wine [19]. These limits are much higher than TCA and TBA.

## 4. Wine Closures

Haloanisoles greatly diminishes the quality of wine, and are characterized by musty and moldy aromas, which can completely overwhelm the sensory profile of the tainted wine [20]. In 1995, Fuller approximated that 2–5% of all wines are affected by ‘cork taint’; however, due to no legal limitations or required reporting of haloanisoles in wine, we can never know how much wine is tainted with haloanisoles. When wine that contains haloanisoles reaches consumers, especially those without an understanding of wine faults, they can reject not only that wine, but the entire brand which produced the wine.

Corks were the first source of TCA contamination documented in 1982. Even though the authors in this first paper detailed the possibility of other sources of contamination, the industry and the literature focused most of their efforts on corks [3].

Wineries have increasingly begun to move away from natural or technical cork closures and have instead turned to screw caps or synthetic closures in order to limit the production of wines with haloanisole taint. By making this shift, wineries are able to better control the amount of haloanisole entering their wines, thus reducing the chances of taint. Additionally, screw caps and synthetic closures are often less expensive than natural or technical cork, allowing wineries to reduce their production costs. A study conducted by the James Halliday Australian Wine Competition found increasing usage of screw caps in Australian wines from 2007 to 2018. They reported 51.5% of wines in 2007 to have used screw caps and 87.9% of wines in 2018 to have used screw caps [21].

Other wineries have declined the use of any closure other than natural cork, fearing consumer rejection. In a case study written about the Rodney Strong Winery and their dilemma over switching from natural cork to either screw or synthetic, they described that “consumers have a great fondness for the ambience and sensuality provided by the cork” [22]. A study conducted in 2007 compared wine closures with consumer perception of wine quality. The researchers compared natural cork, synthetic/plastic, and screwcap and had 106 participants. When asked if “natural corks can sometimes cause wines to smell and taste bad”, only 23.6% of participants believed the statement was true. They found that a moderate number of participants thought that wine closures other than natural cork were used in order to avoid wine spoilage and that most thought using a wine closure other than natural cork was because they were cheaper. A total of 72% of participants thought that synthetic corks were acceptable substitutes, while only 20% thought that screw caps were acceptable [23].

Researchers have conducted many studies to evaluate the efficacy of various enclosures in their capacity of blocking out haloanisoles. One such study, conducted in 2011, compared natural cork, agglomerated, and synthetic closures. Model wine was bottled and sealed with one of the previously mentioned closures, then placed in a 2 L container that could be sealed. Before sealing, deuterated TCA (d_5_-TCA) at a concentration of 64,000 ng in 30 µL of ethanol was added into each 2 L container and then the container was sealed for up to 24 months. Wine was sampled at 0, 3, 6, 12, and 24 months, and it was found that, for the natural and agglomerated corks, the d_5_-TCA stayed in the outer portion of the cork, while the synthetic cork had detectable d_5_-TCA in the model wine starting at month 3 and only increasing till month 24 [24].

Another study conducted in 2013 compared natural cork, agglomerated cork, two types of synthetic cork, and screw cap with saranex liner. White wine was bottled using the previously mentioned closure types, then boxed into cardboard that had been lined with an aluminum bag and three filter papers that contained 183 µg of d_5_-TCA. The aluminum bag was heat-sealed, the cardboard was closed, and wine was stored for either 1, 12, or 30 months before sampling. At month 1, no d_5_-TCA was detected in wine. At month 12, synthetic cork 1 had 1.7 ng/L and screw cap had 2.5 ng/L. At month 30, synthetic cork 1 had 15.3 ng/L, synthetic cork 2 had 3.4 ng/L, and screw cap had an average of 23.5 ng/L. d_5_-TCA was not detected in wines sealed with natural cork or agglomerated [25].

Both studies concluded that natural and agglomerated cork were effective barriers for atmospheric TCA.

The Cork Quality Council (CQC) is a leading authority on cork quality, conducting yearly audits of corks brought in by producers. In 2021, they tested more than 30,000 corks and discovered that the levels of TCA (2,4,6-trichloroanisole) have significantly decreased since 2001, now standing at nearly 99% less than before [26].

The analysis was conducted by ETS laboratories in California, who offer analyses for the detection and quantification of haloanisoles. Even though ETS offers a GC-MS haloanisole panel, the levels of other haloanisoles, such as TeCA, PCA, and TBA have not been reported.

The use of supercritical CO_2_ to extract haloanisoles from cork granules has been a major breakthrough in the industry. This process was initially discovered in 2000, when it was determined to be an effective way to extract TCA from corks. To verify the extraction process, scientists used gas chromatography-mass spectrometry (GC-MS) to test the TCA levels extracted from spiked corks. The results showed that the extracted TCA levels were within 1–4% of the theoretical concentration [27].

Currently, there are various corks available on the market that have been treated with supercritical CO_2_. One such example is DIAM corks, a technical cork which uses this particular process and guarantees that their corks are below quantification for the haloanisoles previously mentioned.

Overall, this extraction process has greatly improved the industry by providing corks that are free of haloanisoles and are much more reliable for winemakers. This process has allowed for a safer and more consistent production of wines without the risk of contamination from TCA, TeCA, PCA, or TBA [28].

## 5. Remediation

Remediation methods for haloanisole-tainted wines focus on fining using a variety of materials. Previously studied materials include molecularly imprinted polymers, ultra-high molecular weight polyethylene, Fibrafix TX-R filter pads, polyaniline, Zeolite-Y molecular sieves, plastic wrap, and yeast hulls [29,30,31,32,33,34,35]. Most treatments, excluding the Fibrafix filter pads, are used in a similar manner: add to the wine, agitate, and then either strain or filter out or allow to settle and rack the wine.

Most remediation methods have only been studied for their effectiveness on decreasing trichloroanisole, while very few studies have focused on removing tribromoanisole. One such study involved the use of polyaniline, an organic polymer, and reported a 75% reduction in tribromoanisole and only 13–15% reduction in trichloroanisole in whiskey. Whiskey was spiked to 20 ng/L TCA and TBA, either 100, 300, or 500 mg/L of the polyaniline materials were added, agitated, then allowed to sit for 1, 8, or 24 h at room temperature. Samples were then filtered and run via GC-MS for haloanisole quantification. Aromatic and phenolic content was maintained [33].

Molecular imprinted polymers, or MIPs, were shown to have recovered 99% of the TCA that was present in the wine. MIPs are “synthetic materials with artificially generated recognition sites able to specifically rebind a target molecule”. Wines already contaminated with TCA were passed through the polymer and then analyzed via GC-MS. Sensory was not conducted to see if there were negative effects on the wine post-treatments [30].

Fibrafix TX-R filters were shown to be able to reduce trichloroanisole to 1.1–1.2 ng/L and tribromoanisole to undetectable levels [31]. In this study, white and rose wines were spiked with TCA and TBA at 5–20 ng/L and then filtered using the TX-R filter pad. Little loss of color and aroma were reported by a sensory panel. While the active ingredient in these filter pads is aluminum silicate, wine filtered with these pads were still observed to be under the maximum legal limit for aluminum [31].

Zeolite-Y molecular sieves are able to “selectively remove TCA from solutions”. These molecular sieves have shown an 87% reduction in TCA in wine. Wine spiked at 20.4 ng/L and then treated with the molecular sieves had a concentration of 2.5 ng/L. Concentrations in wine were determined via gas chromatography-mass spectrometry (GC-MS.) These molecular sieves have the potential to be incorporated into the wine manufacturing process or packaging [29]. In 2016, the European Union (EU) approved the use of filter plates containing zeolites Y-faujasite in order to remove chloroanisoles [36].

Ultra-high molecular weight polyethylene has been shown to be able to treat wine contaminated with TCA to below sensory detection. Ultra-high molecular weight polyethylene or UHMW PE is a synthetic polymer that can remove undesirable flavors and aromas in foods and beverages [32]. In this study, the granular form of the polymer was used. After rinsing it in order to remove any residual ‘plastic’ flavor, the investigators added it to the contaminated wine at a rate of 150 g/L. The contaminated wines were a grenache, shiraz, and chardonnay, all spiked with 80 ng/L TCA. After treatment, the wine was either filtered or racked off. The TCA concentrations post-treatment were 6.4, 6.4, and 2.8 ng/L, respectively. While effective at reducing trichloroanisole levels, UHMW PE was shown to strip wines of color and aromas, which can decrease the overall quality of the wine [32].

Plastic wrap has been reported by consumers to be a quick fix to wine perceived to be “corked”. Research conducted on this topic has shown that it used to be a solution when plastic wrap was made from polyvinyl chloride or PVC. Wine spiked to 9ppt TCA was able to be “cleaned” to below sensory detection for the haloanisole. “The plastic film used is effective in removing HAs in a partially selective manner” [34]. Current plastic wrap that can be purchased at the supermarket is made from low-density polyethylene or LDP and does not have the same effect.

Extrafem yeast hulls developed by Oenobrands have beenshown to effectively reduce TCA, TeCA, and PCA. The yeast hulls undergo a process to increase their ability to absorb odors without imparting negative aromas. With an addition of 400 mg/L of yeast hulls for 48 h and agitating 3 times per day, researchers found a 27% decrease in TCA, 55% decrease in TeCA, and a 73% decrease in PCA. When the treatment was repeated, a 45%, 73%, and 83% decrease was found, respectively. TBA was shown to have a 78% decrease when treated. Sensory conducted on the treated wines showed the elimination of haloanisole smells and no yeast smell or flavor [35].

Table 1 synthesizes haloanisoles and halophenols structures, sources, thresholds, and remediation methods for each.

## 6. Analysis Methodology

Gas chromatography-mass spectrometry (GC-MS) is the industry and academic standard for testing haloanisoles. Due to the haloanisoles being present at such low concentrations and wine being a very complex matrix, sensitive analyses are required.

Table 2 synthesizes eight methods for testing haloanisoles in wine, the atmosphere, and water. These methods all use GC-MS and were considered recent as publication was from 2011 to present. Four out of the six liquid methods use headspace solid phase microextraction (HS-SPME), which allows for little to no sample preparation compared to the liquid-liquid extractions. More current methods also have lower limits of detection (LOD) and limits of quantification (LOQ).

Three analytical methods that do not involve GC-MS use were also found. One study used near infrared spectroscopy, or NIR, to analyze 600 wines of different ages and regions and reported that “these compounds can be determined rapidly and easily with this technique”. NIR was able to detect TCA, TCP, TeCA, TBA, and PCA [45]. Another research group developed a cellular biosensor to detect TCA. This biosensor was able to “detect TCA from cork soaks at concentrations of 1.02–12 ng/L” [46]. The biosensor is highly selective and is only able to detect TCA and no other haloanisoles or halophenols. Lastly, a method for testing corks was developed using “chemical ionization-time-of flight (CI-TOF) mass spectrometry employing the “Vocus” ion source and ion-moleculereactor”. This method only investigated TCA. However, the authors specify that this method, in principle, would be able to detect other haloanisoles and halophenols. This method uses whole corks, does not destroy the cork, and is incredibly fast at 3 s per cork [47].

**Table 2:** GC-MS Methodologies.

Gas Chromatographer	Mass Spectrometer	Column Type	Sample Type	Extraction	Compounds Analyzed	Inlet Temperature	Oven Program	LOD	LOQ	Analysis Time Per Sample	Source
Varian 3800	Varian Saturn 2200 Ion-trap	CP-WAX 52-CB	Wine	Dispersive liquid-liquid microextraction	TCA TBA TeCA PCA	250 °C	35 °C for 1 min 20 °C/min to 170 °C hold for 1 min 3 °C/min to 210 °C hold for 12 min	3 ng/L	10 ng/L	32 min	[48]
Agilent 7890A	Agilent 7000B	DB-5	Wine	HS-SPME 100 µm PDMS	TCA TBA TeCA PCA	280 °C	40 °C for 0 min 30 °C/min to 280 °C hold for 3 min	TCA, TeCA, PCA: Sub ng/L TBA: <1 ng/L	TCA, TeCA, PCA: Sub ng/L TBA: <1 ng/L	11 min	[49]
(2) HP 6890	D1: FID D2: ECD	D1: DB-XLB D2: TG-1301MS	Wine	HS-SPME 100 µm PDMS	TCA TBA TeCA	250 °C	D1: 50 °C for 2 min 20 °C/min to 120 °C 5 °C/min to 250 °C hold for 5 min D2: 50 °C for 20 min 25 °C/min to 85 °C hold for 0.5 min 2 °C/min to 140 °C 40 °C/min to 250 °C hold for 5 min	0.1 ng/L	TCA: 0.4 ng/L TeCA: 0.4 ng/L TBA: 20.5 ng/L	93.75 min	[50]
Agilent 7890A	Agilent 5975TAD	DB-5	Wine	HS-SPME DVB/PDMS	TCA TBA TeCA PCA			TCA: 1.5 ng/L TeCA: 0.5 ng/L PCA: 11.2 ng/L TBA: 7.5 ng/L			[51]
Varian 3800	uECD	VF-5ms	Wine	Vortex assisted liquid-liquid microextraction	TCA TBA TeCA PCA	300 °C	100 °C for 3.5 min 15 °C/min to 115 °C 1 °C/min to 150 °C 25 °C/min to 250 °C hold for 2 min	TCA: 2.1 ng/L TeCA: 2.7 ng/L PCA: 2.9 ng/L TBA: 2.1 ng/L		44.5 min	[52]
Agilent 7890A	Agilent 7000B	HP-5MS HP-35MS	Atmosphere	Tenax GR	TCA TBA TeCA PCA	280 °C	70 °C for 2 min 25 °C/min to 150 °C 3 °C/min to 180 °C 25 °C/min to 300 °C	TCA: 0.6 pg tube-1 TeCA: 0.6 pg tube-1 PCA: 0.4 pg tube-1 TBA: 3.3 pg tube-1	TCA: 2.1 pg tube-1 TeCA: 2.1 pg tube-1 PCA: 1.3 pg tube-1 TBA: 2.2 pg tube-1	25 min	[53]
Agilent 6890 N	Agilent 5973 N	HP-5MS	Atmosphere	Tenax TA	TCA TBA TeCA PCA	250 °C	55 °C for 3 min 15 °C/min to 125 °C 1.5 °C/min to 145 °C 10 °C/min to 183 °C 1.5 °C/min to 195 °C 15 °C/min to 250 °C hold for 3 min	TCA: 0.01 ng tube-1 TeCA: 0.06 ng tube-1 PCA: 0.03 ng tube-1 TBA: 0.05 ng tube-1	TCA: 0.05 ng tube-1 TeCA: 0.1 ng tube-1 PCA: 0.1 ng tube-1 TBA: 0.1 ng tube-1	40 min	[54]
		HP-17MS	Water	HS-SPME PDMS/DVB/CAB	TCA TBA	250 °C	45 °C for 4 min 10 °C/min to 240 °C hold for 1 min 30 °C/min to 280 °C hold for 4 min	TCA: 0.098 ng/L TBA: 0.086 ng/L		30 min	[55]

## 7. Conclusions

Haloanisoles continue to be an issue for wine makers globally. One of the largest issues is the result of the common name of this fault, “cork taint”. This misleading name has caused a complete diversion of research and resources from the other causes of haloanisoles in wine. We believe TBA to be an under-reported fault in the industry due to a lack of knowledge of its vectors. TBP is ubiquitous in the construction industry as a fire retardant. The effect that these compounds have on the quality of wines is not completely understood or described.

As members of the wine and scientific community, it is our duty to transmit to the industry and the public the suggestion to stop referring to haloanisoles in wine as “cork taint”. Instead, we propose “haloanisole taint” (HAT) as a more appropriate name. Future studies into this issue should ideally include all haloanisoles discussed in this review when it comes to sensory and remediation.

## Figures and Tables

**Table 1:** Haloanisoles and halophenols.

Name	Abbreviation	Chemical Structure	Sources *	Thresholds	Remediation
**2,4,6-Trichloroanisole**	TCA	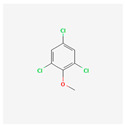 [37]	Corks cleaned with bleach [3] Chlorinated water [4] Chlorine-based cleaners	3–10 ng/L [15,16]	Polyaniline [33] Molecular-imprinted polymers [30] Fibrafix TX-R [31] Zeolite-Y molecular sieves [29] UHMW PE [32] PVC plastic wrap [34] Extraferm Yeast Hulls [35]
**2,4,6-Trichlorophenol**	TCP	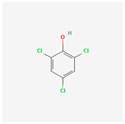 [38]	Biocides containing TCP used to treat cork trees [4]	-	-
**2,4,6-Tribromoanisole**	TBA	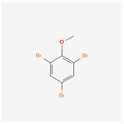 [39]	TBP methylated by fungi	2–7.9 ng/L [6]	Polyaniline [33] Fibrafix TX-R [31] Extraferm Yeast Hulls [35]
**2,4,6-Tribromophenol**	TBP	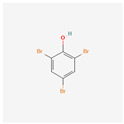 [40]	Fireproofing agents on wood [2,6] spray foam insulation [7] Methyl-bromide fumigant [8]	-	-
**2,3,4,6-Tetrachloroanisole**	TeCA	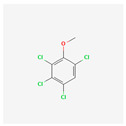 [41]	TeCP containing biocides methylated by fungi	5–15 ng/L [19]	Extraferm Yeast Hulls [35]
**2,3,4,6-Tetrachlorophenol**	TeCP	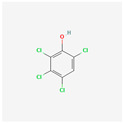 [42]	A major component of PCP biocides [13]	-	-
**Pentachloroanisole**	PCA	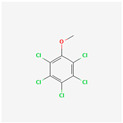 [43]	PCP biocides methylated by fungi	10,000 ng/L [19]	Extraferm Yeast Hulls [35]
**Pentachlorophenol**	PCP	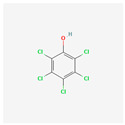 [44]	Biocides containing TCP used to treat cork trees [4]	-	-

* All haloanisoles and halophenols can become aerosolized and resettle onto other surfaces in the winery.

## Data Availability

Not applicable.
